# Role of Physiological Resilience in Aging: Challenges and Opportunities

**DOI:** 10.1093/geroni/igab046.622

**Published:** 2021-12-17

**Authors:** Derek Huffman

**Affiliations:** Albert Einstein College of Medicine, Bronx, New York, United States

## Abstract

Lifespan and healthspan remain a cornerstone of documenting efficacy in aging research. However, it is becoming increasingly appreciated that housing rodents in conventional, unprovoked conditions, rather than exposed to the same variety of stressors normally encountered by free-living humans, has limited our understanding of how these strategies can be translated. Resilience can be defined as the ability of an organism to respond to a physical challenge or stress and return to homeostasis. Indeed, physiologic resilience is recognized to decline with age from a weakening of interactions among multiple physiologic regulatory functions. Here, we have attempted to optimize stress assays as a means of measuring physiologic resilience in mice. Our data demonstrate that these assays can readily detect age-related deficits in recovery, are amendable to geroprotector strategies, including rapamycin, while acute exposure to a stress can accelerate aging and mortality, thereby serving as a potentially useful paradigm for testing age-delaying interventions.

